# The VersaLive platform enables microfluidic mammalian cell culture for versatile applications

**DOI:** 10.1038/s42003-022-03976-8

**Published:** 2022-09-29

**Authors:** Giovanni Marco Nocera, Gaetano Viscido, Stefania Criscuolo, Simona Brillante, Fabrizia Carbone, Leopoldo Staiano, Sabrina Carrella, Diego di Bernardo

**Affiliations:** 1 Telethon Institute of Genetics and Medicine (TIGEM), Via Campi Flegrei 34, 80078 Pozzuoli, NA Italy; 2 Department of Electrical Engineering and Information Technology, University of Naples Federico II, Naples, Italy; 3 CEINGE Biotecnologie Avanzate, Naples, Italy; 4 Department of Chemical, Materials and Industrial Production Engineering, University of Naples Federico II, Naples, Italy; 5 Institute for Genetic and Biomedical Research, National Research Council (CNR), Milan, Italy

**Keywords:** Lab-on-a-chip, Microscopy, Imaging

## Abstract

Microfluidic-based cell culture allows for precise spatio-temporal regulation of microenvironment, live cell imaging and better recapitulation of physiological conditions, while minimizing reagents’ consumption. Despite their usefulness, most microfluidic systems are designed with one specific application in mind and usually require specialized equipment and expertise for their operation. All these requirements prevent microfluidic-based cell culture to be widely adopted. Here, we designed and implemented a versatile and easy-to-use perfusion cell culture microfluidic platform for multiple applications (VersaLive) requiring only standard pipettes. Here, we showcase the multiple uses of VersaLive (e.g., time-lapse live cell imaging, immunostaining, cell recovery, cell lysis, plasmid transfection) in mammalian cell lines and primary cells. VersaLive could replace standard cell culture formats in several applications, thus decreasing costs and increasing reproducibility across laboratories. The layout, documentation and protocols are open-source and available online at https://versalive.tigem.it/.

## Abstract (web summary)

VersaLive is a versatile microfluidic platform with flexible input modes and low-volume media reservoirs that can be used for time-lapse live cell imaging, immunostaining, cell recovery, cell lysis and plasmid transfection.

## Introduction

Micro-scaled systems can be designed to accommodate experimental requirements that are difficult to meet or utterly impossible in standard mammalian cell culture^1–3^. In general, the use of microfluidics for cell culture allows for precise control of the extrinsic factors (e.g., nutrients, drug treatment, sample confinement) while better mimicking physiological conditions^4–7^. Thanks to the reduced volumes, microfluidics allows minimizing the consumption of expensive chemicals. The higher degree of control over the experimental conditions increases the reliability of protocols where the outcome is otherwise more dependent from the operator skills. In the recent years, for instance, the use of microfluidic systems has shown promise in facilitating better cancer diagnosis by standardizing immunohistochemistry biomarker analysis^8–10^. Other recent key applications of microfluidics in mammalian cells are high-throughput single-cell sequencing^11,12^, parallelized drug screening^13^, temporal modulation of treatments^14^, and automated feedback control of biological processes^15^.

Microfluidic platforms are usually designed for specific single applications (e.g., cell culture^4^, single-cell transcriptomics^11^, immunostaining of fixed tissues^9^), and most times involve complex multi-layer microfabrication processes^14,16,17^. Kolnik et al. developed a microfluidic platform for live cell imaging that is able to dynamically stimulate cultivated cells with two inputs and all their mixed ratios^14^. This platform requires a multi-layer master fabrication, uses a complex cell loading protocol, demands the use supplementary equipment (i.e., stepper motors, external controllers) and long connecting tubing, thus imposing dead volumes that exceed the actual volume of the very microfluidic chip. On the other hand, Gagliano et al. exploited a single-layer and self-contained microfluidic device to increase the efficiency of the pluripotency reprogramming process of human somatic cells^4^. This platform has the advantage of a simple geometry and fabrication but with the drawback of the sole static cell culture that limits the possible applications. Both the Kolnik et al. and Gagliano et al. platforms are not optimized for a straightforward recovery of the cultivated cells.

In this work, we designed and developed a versatile microfluidic device, which we named VersaLive, to enable the application of multiple protocols for adherent mammalian cells which include cell culture, immunostaining, live cell imaging and cell retrieval by using standard lab pipettes. To facilitate wide adoption of VersaLive and to make it easy to transfer standard experimental protocols to microfluidics, we designed VersaLive to be replicated via simple microfabrication procedures and so that all the operations on chip can be carried out by standard pipetting. We demonstrated culture of cell lines and primary cells and application of a variety of protocols from immunostaining to plasmid transfection and cell lysis.

## Results and discussion

### The VersaLive design

**Fig. 1:**
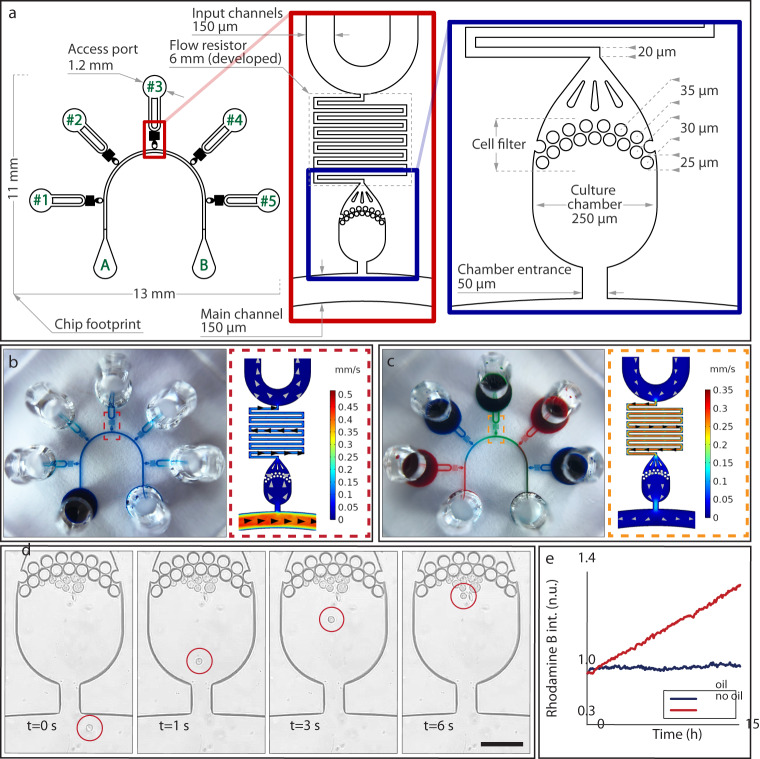
Outline of the VersaLive microfluidic platform. **a** Layout of the microfluidics device with five independent chambers. On one side, all chambers are connected to the main channel that develops from port A to port B of the schematics. Fluids can be directed to each chamber independently by dedicated ports (ports #1 to #5 on the schematics). The serpentine flow resistor prevents shear stress to the cells during the device operations. **b**, **c** Modes of operation of VersaLive and their finite element simulations in COMSOL Multiphysics software (dashed inset). In the “perfusion” single-input mode (**b**), the main channel is exploited to deliver the same medium to all chambers at the same time. Multi-input mode (**c**) exploits the dedicated ports to deliver different inputs (i.e., media, chemicals) to each chamber avoiding cross-contaminations. **d** During cell loading, the cell suspension flows through the main channel and into the chambers. Once in the chamber, cells slow down and are eventually stopped by the filter features as predicted in the velocity profile of the simulation. Scale bar, 150 µm. **e** Solvent evaporation at the reservoirs was observed to increase the solute concentration of the channel content during perfusion. The effect was completely removed by the addition of a layer of mineral oil at the reservoirs. In the plot one single representative run is reported.

The device is shown in Fig. 1; it consists of a gas-permeable elastomer (polydimethylsiloxane (PDMS)) fabricated by means of standard photolithography and soft lithography and bonded onto a microscopy glass coverslip. The device is designed so that only one single photolithographic step is necessary to prepare the wafer thus enabling fast and easy replication of the technology also in laboratories without specific expertise in microfabrication. The PDMS chip manufacturing does not require alignment of layers and the complete fabrication process can be learned by unexperienced personnel over 1 day of training. The necessary material to prepare VersaLive chips is detailed in Supplementary Table 1. As shown in Fig. 1a, the platform consists of five 250 µm-wide culture chambers. The choice over the number of chambers to place on one chip was dictated by the geometry constraints of the 3 mm-wide reservoirs that need to be spaced out within a 30 mm circular coverslip and allow to be cut out using a biopsy punch. Each chamber is connected on one side to a main common channel and, on the other side, to an independent input channel. Each input channel consists of two 150 μm-wide parallel channels to increase robustness against clogging from dust particles. Channels are accessed through ports (Fig. 1a) that act as reservoirs where inputs (e.g., growth medium, drugs, reagents) can be exchanged. Hydrostatic pressure alone drives the flow from the ports to the chambers, thus doing away with pumps, motors, or pressure regulators. Cell filters are embedded at one side of each culture chamber to contain cells within the chamber during the loading phase by preventing anything larger than 5 µm in width to go through the filter. A fluidic resistor 20 μm in width and 6 mm in length connects each culture chamber to its input channels. The suitable fluidic resistance was found testing multiple lengths of the same serpentine design. The resistance has the purpose of allowing cell loading into the chambers within seconds from the filling of the reservoir while ensuring, once the cells adhere to the glass slide, the absence of shear stress during perfusion.

### Use of the microfluidic platform

All operations in VersaLive are carried out by simply pipetting content in and out of the ports that effectively act as reservoirs. VersaLive can be operated in either of two ways: *single-input mode*, where the same input (e.g., cell growth medium) is delivered to all cell chambers (Fig. 1b) or the *multi-input mode*, where a different input can be delivered to each of the 5 chambers (Fig. 1c). Single-input mode is set up by pipetting a volume of up to 20 μL in the reservoir connected to the main channel (port A in Fig. 1b) while leaving all other reservoirs empty. We refer to this configuration as the “*perfusion” single-input mode*, since one constant flow from the filled reservoir to the cell chambers will be present. A variant of the single-input mode is obtained if all reservoirs are filled with the same volume. In this case, no flow will be present in the device, giving rise to a static cell culture. This *“static” single-input mode* of operation is particularly convenient during the cell adhesion phase, when the cells are not fully attached to the chip and a flow would risk displacing them.

**Fig. 2:**
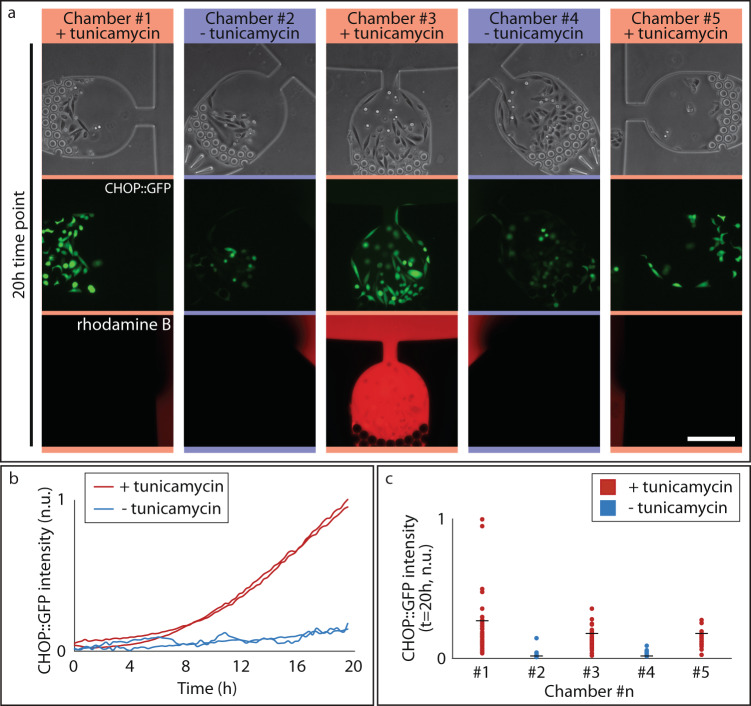
Live cell imaging on VersaLive. **a** CHO-K1 cells in the indicated chambers were exposed to tunicamycin treatment (0.5 μg/mL for 20 h) and the integrated stress response was measured by the intensity of CHOP::GFP fluorescence. The third row shows rhodamine B that was used as flow tracer to check proper flow in the platform throughout the time-lapse acquisition. Scale bar, 150 µm. **b** Time course of the average CHO::GFP fluorescence across cells in chambers #1, #2, #3, #4 (15 min time resolution). **c** Single-cell quantification of the CHO::GFP fluorescence of the images reported in **a** returned a 15-fold difference between the treated and the untreated samples. Dots represents single cells (*n* = 38 in #1, *n* = 28 in #2, *n* = 49 in #3, *n* = 55 in #4, *n* = 24 in #5) while black bars are median values.

In the case of the *multi-input mode*, all input reservoirs are filled while the main channel ports are left empty (Fig. 1c). This configuration creates an active flow across each chamber that it is strong enough to prevent backflow from the main channel, but sufficiently slow to prevent shear stress to the cells (Fig. 2a). We verified that a volume of 20 μL per chamber will suffice for at least 24 h of constant perfusion in a cell incubator. Multi-input mode is applicable, for instance, when different concentrations of a small molecule or antibody need to be tested in parallel, as for drug screening or immunostaining.

COMSOL Multiphysics software was used to simulate each mode of operation (Methods), to compute the resulting velocity profiles when realistic hydrostatic pressure differences are applied across the chip, as shown in the dashed insets (Fig. 1b, c).

Independently from the chosen mode of operation, the cells are always loaded into the chambers by using the “perfusion” single-input mode, as shown in Fig. 1d. Time-lapse acquisition of the cell loading step were used to estimate the cell loading velocity of HeLa cells, which was found to be 150 µm per second at the entrance of the chamber; it then takes an additional 5 seconds to further travel 150 µm before eventually stopping against the filter, in line with the velocity profile obtained in simulations and shown in Fig. 1b, c. Although cells are initially seeded closer to the posts, they subsequently form a uniform monolayer that eventually covers the surface of the chamber. The initial number of cells to load into the chambers may vary according to the cell type and the experimental requirements (e.g., cell size, duration of the experiment, cell coverage, time schedules, necessity of a monolayer, cell line characteristics). The loading rate of cells in a chamber can be easily adjusted by varying the cell suspension concentration. To prevent adhesion of the cells to regions of the chip other than the chambers (e.g., main channel, reservoirs) it is preferable, however, to adjust the cell concentration to keep the loading time short, i.e., within a few minutes. In our experiments, we observed that to carry out the loading of a whole chip within a few minutes, the optimal cell concentration for HeLa cells ranged between 1000 and 5000 cells per microliter.

Exposed to the external environment, the small volume of the reservoirs tends to evaporate over time even if kept in a cell incubator, as shown in Fig. 1e. This effect, however, can be eliminated by pipetting 2.5 μL of mineral oil to the reservoirs, as shown in Fig. 1e where an increase over time of the solute concentration in the reservoir was observed in the absence of oil, but completely abolished in its presence.

### Perfusion cell culture, drug delivery, live cell imaging: stress response quantification in CHO-K1 cells

We validated the ability of the microfluidic platform to deliver independent chemical inputs to each of the five culture chambers by means of a CHOP::GFP tagged CHO-K1 cell line^18^. CHOP is a transcription factor whose expression is induced by the activation of the Integrated Stress Response (ISR) pathway and upon endoplasmic reticulum (ER) stress. Cells were loaded into the chambers and kept overnight in incubator with the static single-input mode in standard growth medium to ensure cell adhesion to the glass surface. The following day, all the reservoirs were emptied by pipetting, and the chip was switched to the multi-input mode of operation by adding fresh medium to the reservoirs connected to chambers #2 and #4, while tunicamycin, dissolved in fresh medium (0.5 µg/mL), was added to chamber #1, #3 and #5, as shown in Fig. 2a. Tunicamycin is a potent inducer of ER stress by blocking N-glycosylation of proteins and thus should induce CHOP::GFP expression in the reporter cells. The VersaLive chip was then placed on an inverted fluorescence microscope and each chamber was imaged for 20 h at 15 min intervals. As shown in Fig. 2a–c, at the end of the treatment, only cells exposed to tunicamycin expressed CHOP::GFP, confirming no cross-flow among chambers. Moreover, as an additional check for the proper function of the device and the absence of cross-flow, we added rhodamine B to the input reservoir of chamber #3. This red fluorescent dye has no toxic effects on cells. As expected, red fluorescence was detected only at the outlet of chamber #3 but was absent in the other four chambers. The expression of the CHOP::GFP over time is reported in Fig. 2b and shows appreciably higher expression in the treated samples already after 8 h of exposure to tunicamycin. To quantify the difference in ER stress among the chambers, the last time point of the treatment was analyzed with single cell resolution. The 20-h tunicamycin exposure resulted in a 15-fold increase in the CHOP::GFP expression (Fig. 2c).

### On-chip immunohistochemistry

**Fig. 3:**
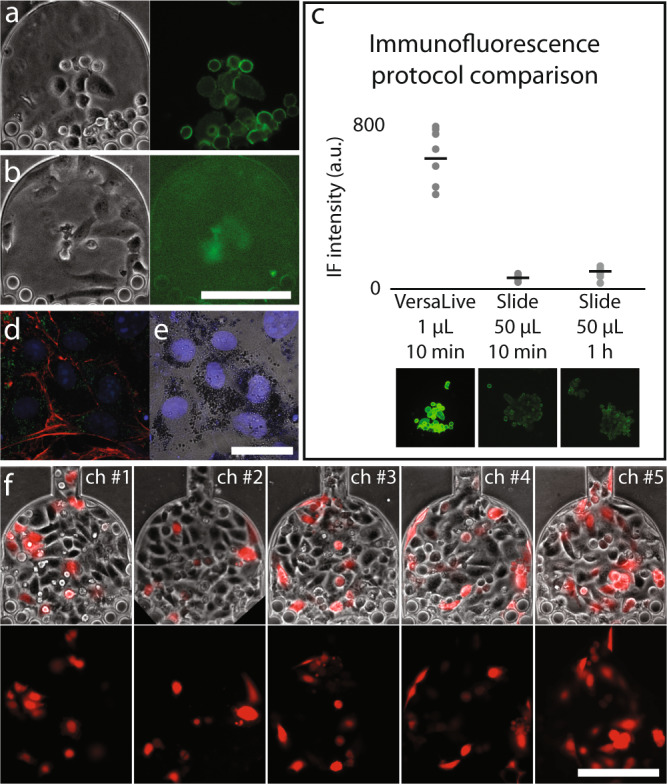
Immunofluorescence (IF) and plasmid transfection on VersaLive. **a**, **b** On-chip immunofluorescence of HER2 membrane receptor on AU565 cells (a, positive control) and HCC38 cells (b, negative control). Scale bar, 150 µm. **c** Comparison of the IF signal intensities for immunofluorescence staining on VersaLive and on slide using a standard protocol. Compared to standard slide protocols, IF on VersaLive resulted in a 10-fold increase in signal while shortening the process time and decreasing reagent consumption. Black bars represent the median values of single ROIs (gray dots, *n* = 8). **d**, **e** Culture of primary mouse retina pigmented epithelium (RPE) cells. On-chip chemical fixation and immunostaining, labeling of citrate synthase (green), f-actin (red), and cell nuclei (blue). The RPE cells were able to adhere to the chip while retaining their characteristic dark pigment. Scale bar, 30 µm. **f** On-chip plasmid transfection of HeLa cells, chamber #1 to chamber #5 (ch#n) of the same chip. Transfection efficiency of 25.3 ± 8.8% (mean ± s. d., *n* = 5). Scale bar, 150 μm.

The single- and multi-input modes of operation can be exploited to implement any protocol of interest such as delivering fixing agents (e.g., paraformaldehyde) and labeled antibodies directly to the cells. Washing and staining steps of established immunostaining protocols can be replicated with the advantage of an effective reagent consumption below one microliter, the gain in signal due to the perfusion and protocol duration cut down from hours to minutes. To showcase this possibility, we performed immunostaining of the human epidermal growth factor receptor 2 (HER2) in two breast cancer cell lines (AU565 and HCC38 as negative control). HER2 is a membrane protein and is over-expressed in some forms of breast cancer, and it is a clinically relevant biomarker for the diagnosis of breast cancer. Cancer cells expressing HER2 (HER2+) are more proliferative but result in a better prognosis while those not expressing HER2 (HER2−) are more resistant to cytotoxic chemotherapy^19^. For this reason, discrimination between these two kinds of tumors is important to decide the therapeutic strategy that will be carried out. Here, HER2+ (AU565) and HER2− (HCC38) breast cancer cell lines were used to validate immunofluorescence protocol in VersaLive. The two types of cells were cultivated on two different VersaLive chips, chemically fixed and fluorescently labeled via anti-HER2 immunostaining directly on the device. As shown in Fig. 3a, b, immunostaining on chip was cell-type specific and resulted in the correct membrane localization of the HER2 receptor. Following quantitative analysis, it is possible to appreciate that staining for 10 min on VersaLive resulted in a 12-fold higher intensity compared to the traditional method on slide. Interestingly, only a small difference in intensity is appreciable when slides are incubated for 1 h instead of 10 min. We associate this increase in signal intensity and image quality to perfusion, that allows for the accumulation of labeled antibodies—therefore signal—over time. This accumulation is not possible in traditional protocols.

### VersaLive is compatible with primary cell culture

Culture of primary cells from patients are of great importance for the diagnosis of diseases or for research purposes, to study primary cells from animal models. VersaLive can be a powerful tool to handle and culture primary cells. As an example, we cultured primary cells collected from the mouse retina pigment epithelium (RPE) cells to demonstrate the possibility of growing primary cells in VersaLive. RPE cells retrieved from fresh tissue were able to adhere to the glass surface of the chip and grow while retaining their characteristic dark pigment (Fig. 3d, e).

We would like to point out that VersaLive, however, is not suited for capturing rare cells. With this purpose, different microfluidic systems have been developed to isolate and concentrate specific types of cells from a bulk sample according to, for instance, their phenotype^20^.

### On-chip plasmid transfection

Liposomal transfection is a well-established chemical method to deliver genes into cells. The interest in the products of the expression of these genes can span from fundamental to medical sciences or for industrial applications^21^. Microfluidics was shown to be a useful tool for increasing the yield of the transfected cells while minimizing the reagent consumption^22^. Previous works exploiting the benefits of perfusion, however, still required external tubing and pumping systems to drive chemicals into the chips^22,23^. The necessity of supplementary hardware increases the cost per experiment and the use of tubing inevitably adds dead volume, meaning that a fraction of the used reagents will not be used for the actual transfection but lost in the process. VersaLive has no dead volumes because it does not require tubing or an external pumping system. In fact, whatever amount of transfection mix that is left in the reservoirs at the end of the perfusion step can be recovered for another on-chip transfection.

We implemented a protocol for on-chip DNA plasmid transfection in VersaLive by adapting lipid-based reagent transfection in 96-well plates to work on chip. From a screening in 96-well plate, we determined the most suitable transfection mix of DNA and lipid complex concentrations. It was found that the optimal combination was 300 ng of DNA and 0.5 μL of transfection reagent (Methods). For the on-chip transfection, DNA and lipids were diluted with cell growth medium to a final volume of 50 μL. Aliquots of this stock transfection mix were used to perfuse the cells over 2 h in multi-input mode with an effective reagent consumption of the order of the few microliters per chamber. In Fig. 3f, we show the result of transfecting cells with a plasmid expressing an mCherry fluorescent reporter downstream of a CMV promoter obtaining a transfection efficiency of 25.3% ± 8.8 per chamber. We then compared the transfection efficiency between VersaLive and standard 96-well plate using the same reverse transfection protocol. The measured transfection efficiency in 96-well plate (27.2 ± 4.6%) and thus comparable to VersaLive.

### Surface coating of VersaLive chips

The coating of the surface prior to the cell seeding is beneficial or, for some cell types, required (e.g., serum-free cultures). VersaLive is fully compatible with any low viscosity coating solution (e.g., poly-L-lysin, vitronectin, fibronectin). As an example of cell culture in VersaLive with coating, we performed live cell imaging the tubulation of transferrin-loaded endosomes in HK2 cells in VersaLive chips coated with a vitronectin solution in PBS (Supplementary Movie 1).

### Cell shear stress can be tuned on VersaLive

Depending on the cell lines studied, shear stress can be a parameter to be either minimized or exploited when culturing under flow. In all experiments showcased in this work, the interest was in minimizing the shear stress on the cells, therefore all pressures used in the protocols fulfill this requirement. However, VersaLive is suitable also for studies where shear stress is a prerequisite. Placing external reservoirs at different heights, it is possible to easily regulate the pressure of the applied flow. For instance, by placing the reservoirs 10 cm above the chip, it was possible to apply a flow of 10 mbar on HeLa cells, which caused the cells to be washed out from the culture chamber (Supplementary Movie 2).

### VersaLive allows post-treatment cell recovery

VersaLive allows direct access to the cells by complete removal of the PDMS from the glass slide where it is reversibly bonded (Supplementary Fig. 1a, b). We envision this feature to be useful for cell re-plating, cell sequencing and all applications that require full recovery of the sample. To demonstrate that the PDMS removal operation does not damage the cells, the same field of view was acquired in live imaging and for the chemically fixed sample, after PDMS removal (Supplementary Fig. 1c, d). Additionally, VersaLive can be used to recover the chamber content in a selective and precise fashion. In Supplementary Fig. 2a–f, we reported the sequence of operations in VersaLive to selectively lyse the content of each chamber starting from chamber #1 to chamber #5. Using the described protocol, it is possible to collect the chamber lysates in separate ports, removing any risk of cross-contamination. By emptying one reservoir at the time or all at once, this same protocol is suitable for a sequential or parallel content recovery.

The operations reported in Supplementary Fig. 2a–f are carried out using dyed solutions to highlight the flow paths. To demonstrate the proper functioning of this protocol, the RNA of the cells from each chamber was collected, a sequencing library was prepared and its quality analyzed by TapeStation instrument to determine its size distribution (Supplementary Fig. 2g). The data show that RNA was successfully recovered from all chambers, despite some differences in the amount of RNA extracted.

## Conclusions

VersaLive offers an easy-to-adopt microfluidic platform for mammalian cells, it enables the choice between perfusion and static cell culture while not requiring any external components to function. VersaLive also allows direct access to the cultured cells with the benefit of reducing the consumption of reagents and plasticware. Additional protocols can be easily adapted to VersaLive by rescaling concentrations used in macroscopic culture system, with the added advantage of reducing costs and speeding up reactions.

Compared to multi-well plate cell culture, VersaLive has several advantages but also some limitations that are schematically summarized in Table 1. VersaLive decreases the consumption of reagents as well as lab plasticware when compared to multi-well plate cell culture. Overall, this translates in a decrease of the cost per experiment. In addition, VersaLive gives to the user the possibility to decide whether to proceed with a traditional static cell culture, similarly to multi-well plates, or to implement a perfusion cell culture, as for more expensive and bulky commercial setups. The implementation of static cell culture in VersaLive, or in microfluidic systems in general, better mimics physiological conditions for cell growth because of the reduced volumes, even compared to multi-well formats (e.g., increase in concentration of secreted factors, optimized geometric constrains), advantages that are not achievable in multi-well plate cell culture. Perfusion cell culture has a set of advantages over static culture methods, such as implementing a continuous flow of nutrients to avoid depletion over time; maintain a constant concentration of a drug or small molecule over time; and speeding up standard protocols.

**Table 1:** Comparison of the main features differing between multi-well plates (96- and 384-well types) and VersaLive.

	96-well plate	384-well plate	VersaLive
Geometric parameters			
Volume, μL	200	75	20 (reservoir) 0.001 (chamber)
Growth area, mm^2^	35	5.6	0.05
Protocol optimization			
Reagent consumption, μL (e.g., antibody for IF)	100	50	1
Protocol speed up (e.g., antibody incubation time)	1–24 h	1–24 h	10 min
IF cost (€ per sample)	1	0.5	<0.01
Cell culture			
Throughput	High	High	Low
Physiological concentration of secreted factors	NO	NO	YES
Perfusion cell culture	NO	NO	YES (1 μL/h)
Stimulus depletion over time (e.g., nutrients, drugs)	YES	YES	NO (perfusion cell culture)
Washout to remove stimulus (e.g., nutrients, drugs)	YES	YES	NO (perfusion cell culture)

Some of the current limitations of VersaLive are its low throughput with up to five chambers per chip, which however could be scaled up by using the same topology but increasing the length of the main channel and thus the number of chambers connected to it. Moreover, the retaining capacity of the cell filters is effective only for cells larger than 5 μm in diameter, while the maximum experiment duration without the intervention of an operator is of about 24 h. However, it is possible to extend the duration of an experiment by using pipette tips attached to the reservoirs to increase the volume capacity. Despite these limitations, we believe that VersaLive provides a unique low-barrier entry point for laboratories interested in adopting microfluidics in their mammalian cell culture activities. Indeed, although there are many microfluidic devices that can be used to perform the single protocols here described, to our knowledge, none of these devices were designed for pipette-based multi-purpose applications. As such, VersaLive offers a unique advantage for those researchers that wish to benefit from the advantages of microfluidic cell culture but have been put off by the complexity, the cost of the necessary equipment and the expertise required. We believe that VersaLive can help “democratize” microfluidics for mammalian cells, thus contributing to increase experimental replicability while minimizing the use of reagents.

The VersaLive technology is open-source and all protocols and materials are available online (https://versalive.tigem.it/).

## Methods

### Design and computer simulations of the device

The VersaLive microfluidic platform was designed using Autodesk Fusion 360. The velocity profile of the chip was modeled using the finite element method (COMSOL Multiphysics 5.4). In the simulated utilization modes (Fig. 2), 0.5 mbar of pressure was applied at every inlet while the outlets were set at zero pressure. This value of pressure was chosen because equivalent to the 5 mm of hydrostatic pressure when the reservoirs are filled to the maximum of their volume capacity. Flow direction in the simulation plots was indicated using arrows whose dimensions reflect the magnitude of the velocity in a logarithmic scale. A 2D CAD model of the chip was exported into Autodesk AutoCAD 2020 to design the printable photolithography mask.

### Fabrication of the VersaLive microfluidic platform

Microfluidic chips were fabricated by using a combination of standard photolithography and soft lithography procedures^24^. The master mold was microfabricated via mask photolithography of SU-8 negative photoresist (SU-8 3035, Kayaku Advanced Materials Inc.) on silicon wafer substrate. The photoresist was spin coated to reach a final thickness of 25 µm and processed following the guidelines of the manufacturer. Feature height was confirmed by measurement via optical microscopy of the cross-section of a silicone replica of the channels. Before the first use of the master, the passivation of its surface is required to facilitate the release step during soft lithography. The master was passivated by vapor deposition of perfluorosilane (1H,1H,2H,2H-perfluorooctyl-trichlorosilane, Merck kGaA). Specifically, the master was placed in a desiccator with a small vial containing 20 µL of perfluorosilane. Vacuum was then applied overnight to allow the perfluorosilane to evaporate and to react with the surface of the silicon wafer, forming a covalently bound super-hydrophobic coating. The passivated master was then used as a mold for the soft lithography part of the fabrication process of the microfluidic device. The VersaLive microfluidic platform is formed by a silicone elastomer (PDMS) bonded to a glass slide. The elastomer base of the PDMS was thoroughly mixed with the curing agent in a 10:1 ratio as reported by the datasheet of the manufacturer (Sylgard 184, Dow Corning). Air bubbles were removed from the uncured polymer applying vacuum to the mix for 2 h or until no bubbles were visible. The mix was then poured onto the master mold for a final thickness of about 5 mm. If required, vacuum was applied a second time to remove the bubbles formed during the pouring step. The mold with the uncured PDMS were then placed in oven at 80 °C for a minimum of 2 h to accelerate the crosslink of the polymer mix. Once cured, the PDMS was peeled off the master mold. The PDMS slab was then placed with the pattern features facing up to cut out the single chips. Similarly, access ports were opened using a 3-mm biopsy punch (ref. 504649, World Precision Instruments). The channels of the PDMS chips were sealed by plasma bonding the chips to round glass cover slide (30 mm in diameter, thickness no. 1, Marienfeld). Glass slides and PDMS chips were first cleaned from dust particles using adhesive tape. For the surface activation of glass slides and PDMS chips, 85 W air plasma at 0.4 mbar or lower (ZEPTO version B, Diener electronic GmbH & Co. KG) were used. For the permanent or reversible bonds, 30 or 10 seconds of air plasma were used, respectively. The chip was then placed in an aluminum lens tube (SM30L05, ThorLabs) used as chip holder. The lens tube allowed the safe transfer of the chip during the experimental workflow (e.g., workbench, incubator, microscope). To allow an easy and reliable image acquisition procedure, the Nikon microscope was equipped with a custom holder for the lens tubes made of 2-mm laser-cut acrylic.

### Cell culture

HeLa WT, together with AU565 and HCC38 breast cancer cell lines were purchased from ATCC and grown in RPMI 1640 (without L-glutamine, EuroClone) cell medium. CHO-K1 reporter cells (kind gift from Prof. David Ron)^18^ were grown in F-12 (Gibco) cell medium. Both cell media were supplemented with 10% fetal bovine serum (FBS, EuroClone), 1% L-glutamine (EuroClone) and 1% penicillin-streptomycin (EuroClone).

Human Proximal tubule epithelial (HK2) cell line was bought from ATCC (#CRL-2190). HK2 cells were cultured in Dulbecco’s Modified Eagle Medium/F12 (DMEM/F12, Gibco) supplemented with 5% FBS, 2 mM L-glutamine, 1 U/ml antibiotics (penicillin/streptomycin), and 1% insulin-transferrin-selenium (ITS-Sigma Aldrich).

Cells were maintained in a cell incubator at 37 °C, 100% humidity and 5% CO_2_ atmosphere. Prior to the loading of a microfluidic device, the content of a T25 flask at 90% confluence was collected according to the following procedure. First, the cell medium in use was removed and the cells were rinsed with 2 mL of phosphate-buffered saline without calcium and magnesium (PBS, EuroClone). To detach the cells from the flask, 0.5 mL of 0.05% trypsin-EDTA (Gibco) were added and the flask was placed back in the incubator for two minutes. To deactivate the trypsin, 2 mL of cell medium was added to the flask and the cells were thoroughly mixed until all visible aggregates were debulked. The resulting cell suspension was directly used for the loading onto the chip. Primary mouse retina pigment epithelium (RPE) were a gift from Sabrina Carrella, PhD (TIGEM) and were obtained as previously described^25^.

### Channel wetting and long-term storage of the chips

The plasma treatment used for the PDMS-to-glass bonding also gives a temporary hydrophilicity to the surface of the channels^24,26^. A hydrophilic channel surface is key for allowing any water-based solution to flow into the microchannels (i.e., wetting). To optimize the fabrication workflow of a batch of chips, the wetting process was carried out within minutes after the bonding procedure. For the wetting of the microfluidic channels, 10 µL of PBS were added in port B of the chip until all channels were filled. If required, all ports were filled with 10 µL of PBS and the chip was placed in a desiccator and vacuum was applied for 15 min or until all air bubbles disappeared. For long term storage (4–6 weeks), all reservoirs of the chip were filled with 20 µL of PBS, the PDMS side of the chips were sealed with office tape (Scotch Magic Tape, 3 M) and the chips were kept at +4 °C until use.

### Loading of the cells onto the microfluidic chip and static cell culture

For the loading of the cells, the PBS used for the wetting of the channels is removed from all reservoirs and 10 µL of cell suspension (1–5 × 10^6^ cells/mL) is added to port B of the device. Upon filling of the reservoir, cells immediately start to flow through the main channel and to enter into the chambers (Fig. 2b). When a given chamber is filled with the suited number of cells, 10 µL of cell medium are added to the respective port to decrease the flow rate across that chamber. When all chambers are filled with cells, 20 µL of cell media are added to port A and port B is emptied to wash the main channel from undesired cells. Port B is then rinsed from the residual cells and filled with 20 µL of cell media. Next, all input ports from #1 to #5 are filled up to a final volume of 20 µL of fresh cell media. An equal volume of cell media in all ports prevents the formation of pressure drops across the chip and enables the static cell culture. Ultimately, 2.5 µL of mineral oil for cell culture (M8410, Merck kGaA) are added to each reservoir to prevent the evaporation of their content. The chips stayed in the incubator overnight at 37 °C, 100% humidity and 5% of CO_2_ atmosphere prior to the beginning of the experiments. The outcomes of the wetting and the cell loading operations were checked using an inverted stereomicroscope (Leica Microsystems).

### Live cell imaging acquisitions

Time lapse acquisitions of CHO-K1 cells were acquired via a Nikon Ti Eclipse microscope equipped with a mercury lamp (Intensilight, Nikon), a EMCCD digital camera (iXon Ultra 897, Andor Technology Ltd) and an incubation chamber (H201-OP R2, Okolab). Prior to the mounting of the chip, the incubator was equilibrated to a temperature of 37 °C and 5% CO_2_ humidified air. Time-lapse acquisitions up to 20 h were acquired using a ×40 air objective (CFI Plan Fluor DLL ×40, 0.75 NA, Nikon Instruments) to collect the highest possible signal. However, because of the off-center position of chamber #5 in this specific device, the acquisition of this chamber with this objective was not achievable in our microscope. For the single-cell analysis, the fluorescence of the cells from all chambers was acquired at the end of the 20 h treatment using a ×20 air objective (CFI Plan Fluor DLL ×20, 0.5 NA, Nikon Instruments). Depending on the experiment, images were collected in phase contrast (PC) and epifluorescence for green, red and yellow wavelengths using the exposure times and filter sets respectively reported in Table 2.

**Table 2:** Summary of the microscopy settings used in this work.

	Exp time (ms)	Ex (nm)	Dm (nm)	Em (nm)
Phase contrast	300	–		–
Chroma EGFP	300	470/40	495	515/30
Nikon TRITC	100	540/25	565	605/55
Nikon FITC	300	480/30	505	535/45

### Stress response assay of CHO-K1 cells under tunicamycin stimulus

After overnight seeding, VersaLive chips with CHO-K1 cells in static culture were removed from the incubator. To each chip, reservoirs A and B were emptied. Next, the content of reservoirs #2 and #4 was renewed with 15 µL of fresh F12 cell medium. Then, the content of reservoirs #1, #3, and #5 was replaced with 15 µL of tunicamycin 0.5 µg/mL in F12 cell medium. Then, 1 µL of sulforhodamine B 0.1 mg/mL (Merck kGaA) was added to reservoir #3 as flow tracer. To all filled reservoirs (#1 to #5), 2.5 µL of mineral oil for cell culture were added to prevent their evaporation. Reservoirs A and B were left empty. The chip was then mounted on the microscope for live cell imaging for a time lapse acquisition of 20 h at intervals of 15 min between each acquisition.

To quantify the stress response of CHO-K1 cells treated and untreated with tunicamycin, live cell microscopy images were analyzed using the Trainable WEKA Segmentation^27^ plugin in the ImageJ software. In brief, the software was trained in distinguishing CHO cells within the culture chambers of VersaLive. The probability map returned by the software was thresholded to be converted in a segmented mask. The mask was then superimposed onto the original image. The average intensities of the resulting single cells were used to quantify their stress response.

### On-chip immunostaining of AU565 and HCC38 breast cancer cells

AU565 and HCC38 breast cancer cell lines were loaded on VersaLive chips and grown overnight in static cell culture as described in a previous section of this work. For the on-chip chemical fixation, after removal of the cell medium, all ports were washed with 20 μL of PBS. Cells were perfused in multi-input mode with paraformaldehyde (PFA, 4%_w/v_ in PBS) for 10 min. Next, all ports were washed twice with 20 μL of PBS followed by 5 min of perfusion of PBS in multi-input mode. To quench the fluorescence of PFA, a blocking solution (50 mM NH_4_Cl; 0.5% BSA in PBS) was perfused in multi-input mode for 30 min. Ultimately, all ports were washed with 20 μL of PBS. For the on-chip immunostaining, anti-HER2 antibody (BB700 Mouse Anti-Human Her2/Neu, BD OptiBuild) diluted 1:100 in blocking buffer was perfused in multi-input for a time defined by the experiment (10 or 30 min). Then, all ports were washed twice with 20 μL of PBS. To preserve the sample, all ports were filled with 20 μL of PBS and 2.5 μL of mineral oil for cell culture to prevent evaporation. Samples were stored at 4 °C.

### On-slide immunostaining of AU565

AU565 (ERBB2+, positive control) and HCC38 (ERBB2−, negative control) breast cancer cell lines were seeded at 60–70% confluency on glass coverslips (EXACTA-OPTECH, area 10 × 10 mm^2^, thickness 0.13–0.16 mm) in 24-well plate.

Following PBS 1× washing, cells were fixed with PFA 4% for 10 min, and then washed with PBS 1×. After fixation, cells were then blocked with blocking buffer, prepared with BSA 0.5% (Sigma-Aldrich) and NH_4_Cl 50 mM (Sigma-Aldrich) in PBS 1×, for 1 h at room temperature (RT). Cells were incubated in humidified environment at RT in the dark with 1:100 primary antibody (BB700-conjugated mouse antihuman-ERBB2 Ab, BD Bioscience). Incubation time was set according to the experimental plan. Primary antibody was prepared in blocking buffer.

Following antibody incubation, cells were washed three times with PBS 1× and once more with ddH_2_O. Then, coverslips were mounted in Fluoroshield^™^ with DAPI (Sigma-Aldrich) and fluorescence images were acquired using a Nikon Ti Eclipse microscope equipped with a mercury lamp (Intensilight, Nikon), a EMCCD digital camera (iXon Ultra 897, Andor Technology Ltd) and a ×20 air objective (CFI Plan Fluor DLL ×20, 0.5 NA, Nikon Instruments).

### On-chip immunostaining of primary mouse RPE cells

Primary mouse RPE cells were loaded on VersaLive chips and let adhere to the microfluidic chip surface overnight in static cell culture, as described in a previous section of this work. On-chip chemical fixation was carried out as previously described for the cancer cell lines. Immunostaining on chip was initiated by perfusing for 10 min the permeabilization buffer (0.3% Triton X-100, 5% FBS in PBS) in multi-input mode. Next, the anti-citrate synthase primary antibody (ab96600, Abcam) in blocking buffer (0.5% BSA, 0.05% saponin, 50 mM NH_4_Cl, 0.02% NaN_3_ in PBS) was perfused in multi-input mode for 10 min. All ports were then washed with 20 μL of PBS. Successively, fluorescently labeled donkey anti-rabbit Alexa Fluor 488 (A-21206, Thermo-Fisher Scientific), Alexa Fluor 568 phalloidin (A-12380, Thermo Fisher Scientific) and DAPI (D1306, Thermo Fisher Scientific) were perfused for 10 min in multi-input mode to stain mitochondria, f-actin and nuclei, respectively. All ports were then washed twice with 20 μL of PBS. To preserve the sample, all ports were filled with 20 μL of PBS and 2.5 μL of mineral oil for cell culture to prevent evaporation. Samples were stored at 4 °C.

Primary mouse RPE cells were imaged via confocal microscopy (LSM 700, ZEISS Microscopy) equipped with 405, 488, 555 nm lasers and a ×40 oil objective (EC Plan-Neofluar ×40/1.30 Oil DIC). For the acquisition, DAPI (blue), FITC (green) and Texas Red (red) filters were used.

### Transferrin recycling assay

VersaLive chips were coated with truncated human recombinant vitronectin (5 μg/mL in PBS 1×) using the chip in multi-input mode for 30 min at room temperature prior to cell loading. After cell loading on chip, cells were let adhere overnight in incubator (37 °C, 5% CO_2_). At the moment of the experiment, HK2 cells were perfused in multi-input mode at 37 °C for 30 min with serum-free medium with 50 μg/mL Alexa Fluor 488-conjugated transferrin (T13342, Thermo Fisher Scientific) to allow endocytosis and loading of recycling endosomes. Then the medium was changed and serum-free medium with 50 μg/mL unlabeled transferrin was added to allow the recycling of Alexa Fluor 488-conjugated transferrin that was evaluated by live imaging by using a Nikon Ti microscope equipped with a spinning disk module and a ×100 1.5 NA oil objective.

### On-chip plasmid transfection

For the on-chip transfection, HeLa cells were loaded one day prior to transfection following the already described procedure. The transfection mix (Lipofectamine™ 3000 Transfection Reagent, ThermoFisher Scientific) was prepared according to the manufacturer guidelines and optimized to the VersaLive volumes. The mix contained 300 ng of DNA, 0.5 μL of Lipofectamine reagent, 0.6 μL of P3000 reagent and 10 μL of Opti-MEM medium. This mix was then diluted in complete growth medium to a final volume of 50 μL. The cells were perfused via multi-input mode in a cell incubator for 2 h adding 5 μL of diluted transfection mix in each chamber port and mineral oil to prevent their evaporation. After transfection, the mix was replaced by complete growth medium and the chip was left in static single-input mode in a cell incubator for 24 h. Then, images were acquired using the Nikon Ti Eclipse microscope.

### Multi-well plasmid reverse transfection

Hela cells were reversely transfected with pCMV-NLS-mcherry-ccp3 obtained from Golden gate cloning (EMMA toolkit) and an empty vector using Lipofectamine 3000 (ThermoFisher Scientific) according to the manufacturer’s instructions. Twenty-four hours post-transfection, cells were harvested to measure fluorescence via fluorescence activated cell sorting (FACS) analysis.

### Evaporation of the inlet reservoirs

The effect of the evaporation at the inlet reservoirs was assessed by running two different microfluidic devices in multi-input mode. Input reservoirs #1, #2, #4 and #5 were filled with 25 µL of DI water; input reservoir #3 was filled with 25 µL of rhodamine B (0.1 mg/mL). For one of the two devices, 2.5 µL of mineral oil for cell culture were added to the inlet reservoirs to prevent their evaporation. The intensity of rhodamine B was measured within the culture chamber over 15 h at 15 min intervals. Measurements were performed at the same experimental conditions used for live cell microscopy experiments. Data was processed using ImageJ software.

### PDMS removal upon reversible bonding

To facilitate the removal of the PDMS, a single-edge razor blade was gently inserted at the interface between the PDMS and the glass slide all along the perimeter of the chip (Supplementary Fig. 1a). Successively, the PDMS chip was pinched to peel it off the glass slide that sealed the channels (Supplementary Fig. 1b). For the out-chip chemical fixation, the PDMS chip was first removed from the glass slide. Then, the glass slide with the exposed cells was rinsed with PBS and later immerged in the 4%_wt_ PFA solution for 30 min at room temperature. The slide was then rinsed with PBS and gently dried.

### Chip content recovery for RNA extraction

Poly(A)-RNA was captured using poly(T)-resin microparticles, as previously described by the authors in refs. 11,28. The lysis buffer, with the composition described in literature^28^, was diluted 1:1 in PBS 1× prior to start the experiments. Before each extraction run, a stock suspension of beads in lysis buffer (3000 beads/μL) was prepared.

The sequence for recovering the chip content starts with the chip in multi-input mode (10 μL per reservoir, Supplementary Fig. 2a). Next, port A is filled with lysis buffer (7.5 μL). In this configuration, the lysis buffer is able to flow through the main channel but not to enter the chambers. To start the lysis of a chamber, however, it is sufficient to empty the corresponding chamber reservoir (Supplementary Fig. 2b–f). To collect the poly(A)-RNA and preserve it from degradation, an aliquot of beads (3 μL) was placed at each port (#1 to #5). This amount was sufficient to cover the bottom surface of the port without creating backpressure. After 10 min, no cells were still visible in the chambers and the lysis process was considered completed.

Resin microparticles were recovered from each chamber reservoir (#1 to #5) and put into 200 μL tubes. The microparticles were then washed twice with 100 μL of SSC 6X and once with 100 μL of RT buffer. All centrifugation steps were performed at 1000 × *g* for 1 min. The reverse transcription, the exonuclease, PCR and cDNA library purification steps were performed as reported in literature^28^. The amplified cDNA was purified with the Ampure XP bead protocol. A 0.6× (sample volume/Ampure XP beads volume) volume of Ampure XP beads was added to each sample. Finally, the cDNA was eluted in 10 μL of RNAse-free water. Quantitative and qualitative analysis of each sample was performed using a TapeStation D5000 high sensitivity chip.

### Statistics and reproducibility

Image analysis and data processing was performed using ImageJ and Microsoft Excel and error bars represent median ± SD unless otherwise noted. Replicates are biological, representing experiments on the same cell line but performed on different days. Experiments were repeated at least three times. Sample size varied depending on methodology and is defined in figure legends.

### Reporting summary

Further information on research design is available in the Nature Research Reporting Summary linked to this article.

## Supplementary information

Supplementary Material

Description of Additional Supplementary Files

Supplementary Data 1

Supplementary Data 2

Supplementary Movie 1

Supplementary Movie 2

Reporting Summary

## Data Availability

The printable design for the OkoLab H101 stage incubator adapter for the Nikon Ti light microscope is provided as “Supplementary Data 1.dxf.” All raw and processed data generated during and analyzed during the current study (e.g., images, tables, segmentation masks) is provided as “Supplementary Data 2.zip.” The transferrin-loaded endosome tubulation in HK2 cells is provided as “Supplementary Movie 1.avi.” The effect of high shear stress on HeLa cells on chip is provided as “Supplementary Movie 2.avi.”
