# Author Correction: The VersaLive platform enables microfluidic mammalian cell culture for versatile applications

**DOI:** 10.1038/s42003-022-04059-4

**Published:** 2022-10-13

**Authors:** Giovanni Marco Nocera, Gaetano Viscido, Stefania Criscuolo, Simona Brillante, Fabrizia Carbone, Leopoldo Staiano, Sabrina Carrella, Diego di Bernardo

**Affiliations:** 1 Telethon Institute of Genetics and Medicine (TIGEM), Via Campi Flegrei 34, 80078 Pozzuoli, NA Italy; 2 Department of Electrical Engineering and Information Technology, University of Naples Federico II, Naples, Italy; 3 CEINGE Biotecnologie Avanzate, Naples, Italy; 4 Department of Chemical, Materials and Industrial Production Engineering, University of Naples Federico II, Naples, Italy; 5 Institute for Genetic and Biomedical Research, National Research Council (CNR), Milan, Italy

**Keywords:** Lab-on-a-chip, Microscopy, Imaging

Correction to: *Communications Biology* 10.1038/s42003-022-03976-8, published online 29 September 2022.

In this article the affiliation details for Diego di Bernardo were incorrectly given as CEINGE Biotecnologie Avanzate, Naples, Italy but should have been Department of Chemical, Materials and Industrial Production Engineering, University of Naples Federico II, Naples, Italy. The original article has been corrected.

