# Supplementary material for: The VersaLive platform enables microfluidic mammalian cell culture for versatile applications
Source: Commun Biol. 2022 Sep 29;5:1034. doi: 10.1038/s42003-022-03976-8 (PMC9522807; doi:10.1038/s42003-022-03976-8)
Supplement: Supplementary file 1 — Supplementary Material [file 42003_2022_3976_MOESM1_ESM.pdf]

The VersaLive platform enables microfluidic mammalian cell culture for versatile applications – Supplementary Material

Authors: Nocera Giovanni Marco[1,2,3], Viscido Gaetano[1,4], Criscuolo Stefania[1], Brillante Simona[1], Carbone Fabrizia[1], Staiano Leopoldo[1,5], Carrella Sabrina[1], di Bernardo Diego[1,3] *

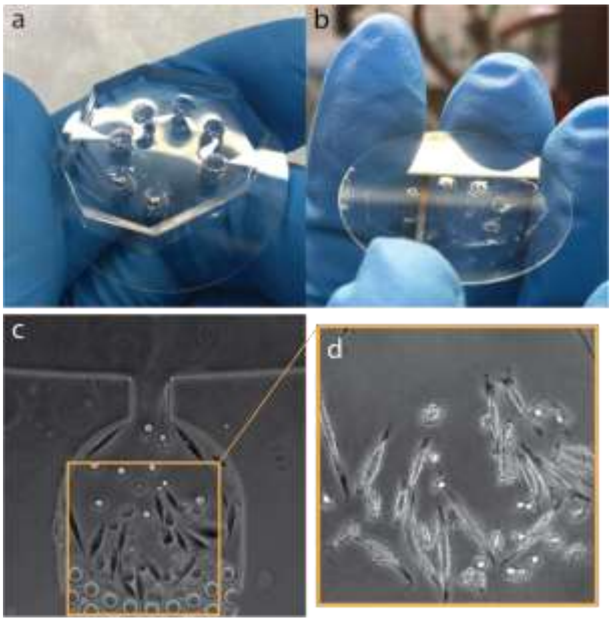

Supplementary Figure 1: *(a, b) Opening of the chip for direct access to the cultivated cells. (c, d) On-chip chemical fixation of previously live-imaged cells and PDMS chip removal. PDMS removal does not affect the cell position of on-chip chemically fixed CHO-K1 cells respect to the live imaged sample. We envision this feature to enable cell re-plating, cell sequencing and all applications that require full recovery of the sample. Panel (c) is 410 µm in width.*

[1] Telethon Institute of Genetics and Medicine (TIGEM), Via Campi Flegrei 34, 80078 Pozzuoli (NA), Italy
[2] Department of Electrical Engineering and Information Technology, University of Naples Federico II, Naples, Italy
[3] CEINGE Biotecnologie Avanzate, Naples, Italy
[4] Department of Chemical, Materials and Industrial Production Engineering, University of Naples Federico II, Naples, Italy
[5] Institute for Genetic and Biomedical Research, National Research Council (CNR), Milan, Italy
* Corresponding author

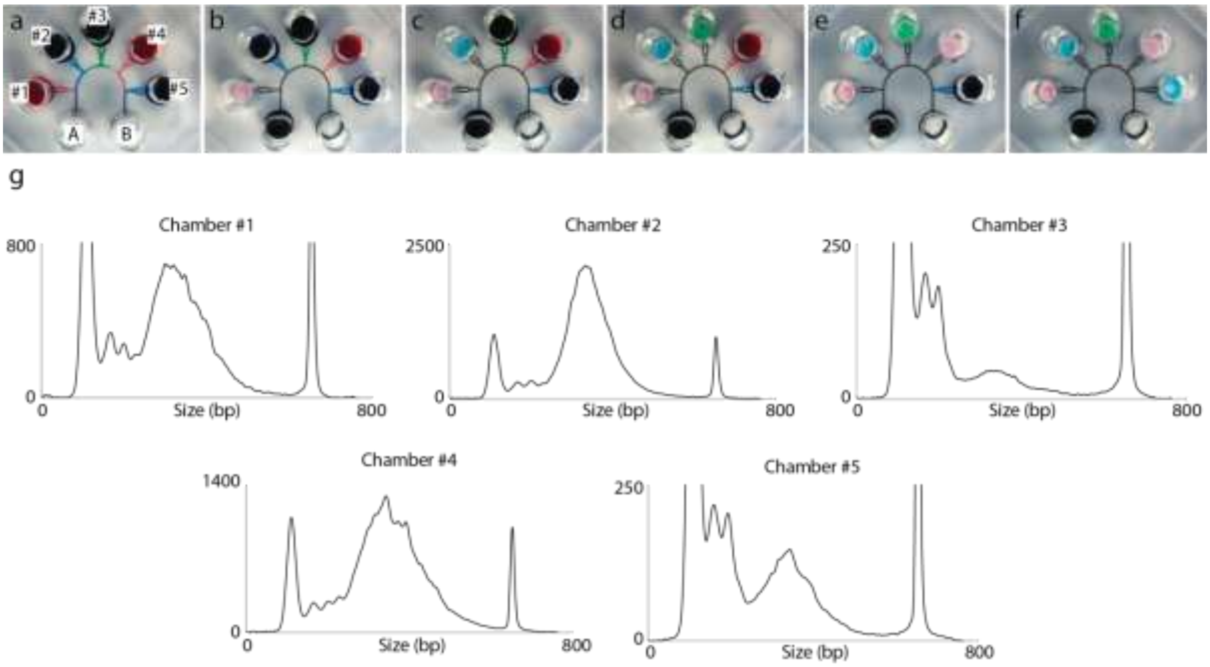

Supplementary Figure 2: *RNA extraction from VersaLive chip. (a-f) Operation sequence of the protocol for RNA extraction from each chamber that prevents cross-contaminations. (g) RNA analysis and quantification of the cDNA library extracted from each chamber of a same chip.*

Supplementary Table 1 List of required materials and equipment for producing VersaLive chips in the lab.

**Item name**	**Item description, link**
VersaLive master	Mold for replicating VersaLive PMDS chips. Master fabrication requires a cleanroom environment but masters can also be purchased from specialized companies or facilities.
PDMS kit	Material for the fabrication of the microfluidic chips via soft-lithography (Dow SYLGARD™ 184 Silicone Elastomer Kit)
Trichloro(1*H*,1*H*,2*H*,2*H*-perfluorooctyl)silane	Fluorosilane for master passivation (CAS number: 78560-45-9)
Vacuum desiccator	For the degassing of the uncured PDMS as well as the passivation of the master

Glass slides, 30 mm diameter, #1 thickness	Slides for the sealing of the bottom side of the chips (Marienfeld, ref. 0111700)
Plasma surface activator (chamber or corona discharge wand)	Plasma activation is required to bond the PDMS to the glass slides. It can be achieved by using plasma chambers but also cheaper corona discharge wands.
Tweezers, fine tip	Tweezers are useful to handle all components (Bahco, ref. TL AA-SA SL)
Biopsy punch	Punches are required to open the access ports of the chips. A diameter of 3 mm is suitable for most applications but other sizes can be used too (World Precise Instrument, ref. 504649)
Single-edge razor blades	To cut chips out of the PDMS slabs (Personna, ref. PSA660210)
[Optional] Scalpel	A curved blade is particularly useful to free the PDMS slab from the master mold (Swann-Morton, blade n. 12)
[Optional] Chip holder	Useful tool to carry the chips without the risk of damaging them (ThorLabs, ref. SM30L05)
[Optional] Chip holder support	Adapter for OkoLab H101 stage incubator for Nikon Ti light microscope (custom made, 2 mm laser cut acrylic). See Supplementary File 1
