# Supplementary material for: The VersaLive platform enables microfluidic mammalian cell culture for versatile applications
Source: Commun Biol. 2022 Sep 29;5:1034. doi: 10.1038/s42003-022-03976-8 (PMC9522807; doi:10.1038/s42003-022-03976-8)
Supplement: Supplementary file 2 — Description of Additional Supplementary Files [file 42003_2022_3976_MOESM2_ESM.pdf]

Description of Additional Supplementary Files

File Name: Supplementary Data 1

Description: Adapter for OkoLab H101 stage incubator for Nikon Ti light microscope

File name: Supplementary Data 2

Description: The source data behind all Figures and organized by Figure number

File Name: Supplementary Movie 1

Description: Transferrin-loaded endosome tubulation in HK2 cells

File Name: Supplementary Movie 2

Description: Shear stress generated on chip disrupts HeLa cells
