# Supplementary material for: The VersaLive platform enables microfluidic mammalian cell culture for versatile applications
Source: Commun Biol. 2022 Sep 29;5:1034. doi: 10.1038/s42003-022-03976-8 (PMC9522807; doi:10.1038/s42003-022-03976-8)
Supplement: Supplementary file 7 — Reporting Summary [file 42003_2022_3976_MOESM7_ESM.pdf]

nature portfolio

Corresponding author(s): Diego di Bernardo

Last updated by author(s): Sep 2, 2022

Reporting Summary

Nature Portfolio wishes to improve the reproducibility of the work that we publish. This form provides structure for consistency and transparency in reporting. For further information on Nature Portfolio policies, see our Editorial Policies and the Editorial Policy Checklist.

Statistics

For all statistical analyses, confirm that the following items are present in the figure legend, table legend, main text, or Methods section.

n/a	Confirmed	
☐	☒	The exact sample size (*n*) for each experimental group/condition, given as a discrete number and unit of measurement
☐	☒	A statement on whether measurements were taken from distinct samples or whether the same sample was measured repeatedly
☐	☒	The statistical test(s) used AND whether they are one- or two-sided<br>*Only common tests should be described solely by name; describe more complex techniques in the Methods section.*
☒	☐	A description of all covariates tested
☐	☒	A description of any assumptions or corrections, such as tests of normality and adjustment for multiple comparisons
☐	☒	A full description of the statistical parameters including central tendency (e.g. means) or other basic estimates (e.g. regression coefficient) AND variation (e.g. standard deviation) or associated estimates of uncertainty (e.g. confidence intervals)
☐	☒	For null hypothesis testing, the test statistic (e.g. *F*, *t*, *r*) with confidence intervals, effect sizes, degrees of freedom and *P* value noted<br>*Give P values as exact values whenever suitable.*
☒	☐	For Bayesian analysis, information on the choice of priors and Markov chain Monte Carlo settings
☒	☐	For hierarchical and complex designs, identification of the appropriate level for tests and full reporting of outcomes
☒	☐	Estimates of effect sizes (e.g. Cohen's *d*, Pearson's *r*), indicating how they were calculated

Our web collection on statistics for biologists contains articles on many of the points above.

Software and code

Policy information about availability of computer code

Data collection	ImageJ Trainable WEKA Segmentation plugin
Data analysis	ImageJ, Microsoft Excel

For manuscripts utilizing custom algorithms or software that are central to the research but not yet described in published literature, software must be made available to editors and reviewers. We strongly encourage code deposition in a community repository (e.g. GitHub). See the Nature Portfolio guidelines for submitting code & software for further information.

Data

Policy information about availability of data

All manuscripts must include a data availability statement. This statement should provide the following information, where applicable:

- Accession codes, unique identifiers, or web links for publicly available datasets
- A description of any restrictions on data availability
- For clinical datasets or third party data, please ensure that the statement adheres to our policy

All raw and processed data generated during and analysed during the current study (e.g., images, tables, segmentation masks) will be provided as Supplementary Material. All file formats can be opened using free software (e.g., ImageJ, VLC, OpenOffice) except for the physics simulation files that require COMSOL software and cannot be converted. No custom primers were used in this manuscript. PCR was used only to obtain cDNA using random priming and we cited the relevant literature for the detailed protocol.

Field-specific reporting

Please select the one below that is the best fit for your research. If you are not sure, read the appropriate sections before making your selection.

☒ Life sciences ☐ Behavioural & social sciences ☐ Ecological, evolutionary & environmental sciences

For a reference copy of the document with all sections, see nature.com/documents/nr-reporting-summary-flat.pdf

Life sciences study design

All studies must disclose on these points even when the disclosure is negative.

Sample size	Sample size was determined by the size of the microfluidic chambers where cells were cultivated.
Data exclusions	During a 20 hour live cell microscopy acquisition, the field of view of one chamber of the chip resulted off centered and we were unable to include it in the time lapse at 40x magnification. However, all fields included this one were imaged at the end of the treatment using a 20x objective.
Replication	All replications of the experiments presented in this work were successful.
Randomization	Randomization was not implemented because not relevant. All experiments were performed with their control on the same microfluidic chip to compare the biological response of the samples.
Blinding	Blinding was not applicable in this work as its focus was the development of a novel microfluidic platform.

Reporting for specific materials, systems and methods

We require information from authors about some types of materials, experimental systems and methods used in many studies. Here, indicate whether each material, system or method listed is relevant to your study. If you are not sure if a list item applies to your research, read the appropriate section before selecting a response.

Materials & experimental systems

n/a	Involved in the study	
☐	☒	Antibodies
☐	☒	Eukaryotic cell lines
☒	☐	Palaeontology and archaeology
☒	☐	Animals and other organisms
☒	☐	Human research participants
☒	☐	Clinical data
☒	☐	Dual use research of concern

Methods

n/a	Involved in the study	
☒	☐	ChIP-seq
☒	☐	Flow cytometry
☒	☐	MRI-based neuroimaging

Antibodies

Antibodies used	Mouse Anti-Human Her2/Neu (BB700, BD OptiBuild); fluorescently labeled donkey anti-rabbit Alexa Fluor 488 (A-21206, Thermo-Fisher Scientific); Alexa Fluor 568 phalloidin (A-12380, Thermo Fisher Scientific).
Validation	BB700, BD OptiBuild:<br>Fendly BM, Kotts C, Vetterlein D, et al. The extracellular domain of HER2/neu is a potential immunogen for active specific immunotherapy of breast cancer. J Biol Response Mod. 1990; 9(5):449-455. (Biology).<br><br>Gullick WJ. The role of the epidermal growth factor receptor and the c-erbB-2 protein in breast cancer. Int J Cancer. 1990; 5:55-61. (Biology).<br><br>Stancovski I, Hurwitz E, Leitner O, Ullrich A, Yarden Y, Sela M. Mechanistic aspects of the opposing effects of monoclonal antibodies to the ERBB2 receptor on tumor growth.. Proc Natl Acad Sci USA. 1991; 88(19):8691-5. (Biology).<br>Shalaby MR, Shepard HM, Presta L, et al. Development of humanized bispecific antibodies reactive with cytotoxic lymphocytes and tumor cells overexpressing the HER2 protooncogene.. J Exp Med. 1992; 175(1):217-25. (Biology).<br><br>Szollosi J, Balazs M, Feuerstein BG, Benz CC, Waldman FM. ERBB-2 (HER2/neu) gene copy number, p185HER-2 overexpression, and intratumor heterogeneity in human breast cancer. Cancer Res. 1995; 55(22):5400-5407.<br><br>A-21206, Thermo-Fisher Scientific:<br>A population of glomerular glutamatergic neurons controls sensory information transfer in the mouse olfactory bulb (Nature Communications, 2014) - Authors: Tatti R,Bhaukaurally K,Gschwend O,Seal RP,Edwards RH,Rodriguez I,Carleton A<br>"A-21206 was used in immunohistochemistry to analyze glomerular glutamatergic neurons and their control of sensory

information transfer in the mouse olfactory bulb"

Parental haplotype-specific single-cell transcriptomics reveal incomplete epigenetic reprogramming in human female germ cells (Nature Communications, 2018) - Authors: Vértesy Á,Arindrarto W,Roost MS,Reinius B,Torrens-Juaneda V,Bialecka M,Moustakas I,Ariyurek Y,Kuijk E, Mei H,Sandberg R,van Oudenaarden A,Chuva de Sousa Lopes SM
"A-21206 was used in Immunohistochemistry-immunofluorescence to demonstrate that germ cells from various stages monoallelically express imprinted genes and confirm this by methylation patterns."

A-12380, Thermo Fisher Scientific:
A high-throughput, cell-based screening method for siRNA and small molecule inhibitors of mTORC1 signaling using the In Cell Western technique.
Hoffman GR, Moerke NJ, Hsia M, Shamu CE, Blenis J,
Assay Drug Dev Technol (2010) 8:186-199

A mutant alphaII-spectrin designed to resist calpain and caspase cleavage questions the functional importance of this process in vivo.
Meary F, Metral S, Ferreira C, Eladari D, Colin Y, Lecomte MC, Nicolas G
J Biol Chem (2007) 282:14226-14237

A requirement for cytoplasmic dynein and dynactin in intermediate filament network assembly and organization.
Helfand BT, Mikami A, Vallee RB, Goldman RD
J Cell Biol (2002) 157:795-806

Eukaryotic cell lines

Policy information about cell lines

Cell line source(s)	HeLa WT, together with AU565 and HCC38 breast cancer cell lines were purchased from ATCC
Authentication	Cell authentication was provided by ATCC
Mycoplasma contamination	All cell lines were tested negative for mycoplasma contamination
Commonly misidentified lines (See ICLAC register)	*Name any commonly misidentified cell lines used in the study and provide a rationale for their use.*
